# Antimicrobial Susceptibility, and Molecular Characterization of *Staphylococcus aureus* Isolated From Different Raw Milk Samples in China

**DOI:** 10.3389/fmicb.2022.840670

**Published:** 2022-05-13

**Authors:** Huimin Liu, Lei Dong, Yankun Zhao, Lu Meng, Jiaqi Wang, Cheng Wang, Nan Zheng

**Affiliations:** ^1^Ministry of Agriculture Laboratory of Quality and Safety Risk Assessment for Dairy Products (Beijing), Institute of Animal Science, Chinese Academy of Agricultural Sciences, Beijing, China; ^2^Ministry of Agriculture—Milk and Dairy Product Inspection Center (Beijing), Beijing, China; ^3^Institute of Quality Standard and Testing Technology, Xinjiang Academy of Agricultural Sciences, Urumqi, China

**Keywords:** antimicrobial resistance, virulence genes, *Staphylococcus aureus*, public health, raw milk

## Abstract

*Staphylococcus aureus* (*S. aureus*) is one of the main pathogens in different raw milk and dairy products, which may lead to economic losses. *Staphylococcus aureus* is a significant and costly public health concern because it may enter the human food chain and contaminate milk causing foodborne illness. This study aimed to investigate the prevalence, antimicrobial susceptibility and virulence genes of *S. aureus* in raw milks. In total, 125 raw milk samples collected from goat (*n* = 50), buffalo (*n* = 25), camel (*n* = 25), and yak (*n* = 25) were collected from 5 provinces in China in 2016. Out of 125 samples, 36 (28.8%) *S. aureus* were isolated (16 from goat, 9 from buffalo, 6 from camel, and 5 from yak). Out of 36 *S. aureus*, 26 strains (26/36, 72.2%) showed antibiotics resistance, and 6 strains isolated from goats were identified as methicillin-resistant *S. aureus* (MRSA). The antimicrobial resistance against Penicillin G, tetracycline and gentamicin was 50% (18/36), 41.7% (15/36), and 36.1% (13/36), respectively. 19 *S. aureus* (52.8%) were considered as multidrug resistant. The highest prevalence of resistant *S. aureus* was observed in goat milk (13/36, 36.1%). Among the 36 strains, 16 isolates harbored three or more resistant genes. The resistance genes were detected in 25 *S. aureus*, including 13 strains in goat, 5 strains in buffalo, 4 strains in camel, and 3 strains in yak. Among the 26 resistant strains, 61.5% of isolates harbored three or more resistant genes. The resistance genes were detected in 25 *S. aureus*, including 13 strains in goat milk, 5 strains in buffalo milk, 4 strains in camel milk, and 3 strains in yak milk. The most predominant resistance genes were *bla*Z (18/26, 69.2%), *aac6*′-*aph2″* (13/26, 50.0%), and *tet*(M) (10/26, 38.5%). The *mec*A, *ant(6)-*Ia and *fex*A gene were only detected in *S. aureus* from goat milk. The most predominant toxin gene were *sec* (8/26, 30.8%). The majority of *S. aureus* were multidrug resistant and carried multiple virulence genes, which may pose potential risk to public health. Our findings indicated that the prevalence and antimicrobial resistance of *S. aureus* was a serious concern in different raw milks in China, especially goat milks.

## Introduction

*Staphylococcus aureus* (*S. aureus*) is one of the main pathogens in mastitis of involved in intramammary infections in cows, goats, and sheep, which may lead to economic losses due to reduced milk production and poor milk quality (Aires-de-Sousa et al., 2007; Stapels et al., 2014). Contamination of raw milk with *S. aureus* has a highly potentially hazardous in China (Chao et al., 2015; Lan et al., 2017; Liu et al., 2017). *Staphylococcus aureus* can cause specific toxin-mediated conditions, such as scalded skin syndrome, staphylococcal food poisoning and toxic shock syndrome (Schmidt et al., 2017). Risk assessment for *S. aureus* in different raw milk samples should been conducted (Cortimiglia et al., 2015; Xu et al., 2015; Ding et al., 2016).

*Staphylococcus aureus* infections are related to the expression of virulence factors. Many potential virulence factors, such as enzymes and exotoxins, contribute to cause *S. aureus* diseases. *Staphylococcus aureus* can harbor different virulence genes encoding for enterotoxins, enterotoxin-like exfoliative toxin, toxic shock syndrome toxin-1 (TSST-1), and Panton-Valentine leukocidin (PVL) (Kot et al., 2016; Wang et al., 2016). Thermostable staphylococcal enterotoxins generally retain their biological activity after pasteurization treatment (Guimaraes et al., 2013). *Staphylococcus aureus* with toxin genes were present in about 60.0% of goat, ovine, caprine, and bubaline origin (Mork et al., 2010; Carfora et al., 2015). An efficient screening to detect the virulence genes in different raw milks is necessary.

The use of antibiotics in veterinary practice could cause the selection of antibiotic resistant *S. aureus*, which is a public health problem (Gomes and Henriques, 2016). *Staphylococcus aureus* is frequently resistant to antibiotics because it produces exopolysaccharide which forms a barrier to bacterial cell penetration by several antimicrobial agents (Johnston, 1998). Moreover, *S. aureus* can also develop acquired resistance to many other antimicrobial agents by carrying various resistance traits on plasmids or transposons (Chajecka-Wierzchowska et al., 2015). Some studies have reported that *S. aureus* which originated from goats, buffaloes, camels, and yak milks, showed high antimicrobial resistance patterns (Pamuk et al., 2012; Biswas et al., 2014; Cortimiglia et al., 2015; Redwan et al., 2016). Therefore, monitoring antimicrobial resistance in *S. aureus* from different raw milks is necessary to predict the risk on antimicrobial resistance of *S. aureus*, and to make decisions regarding antibiotic treatments on animals from a pre-harvest food safety standpoint.

The antimicrobial resistance and virulence characteristics of *S. aureus* from cow milk samples are well described. However, data regarding *S. aureus* from different raw milk samples in China are relatively limited. Therefore, the present work aimed to determine the prevalence, antimicrobial susceptibility and key virulence genes of *S. aureus* isolated from goat, buffalo, camel, and yak milk samples in China.

## Materials and Methods

### Collection of Samples

**FIGURE 1:**
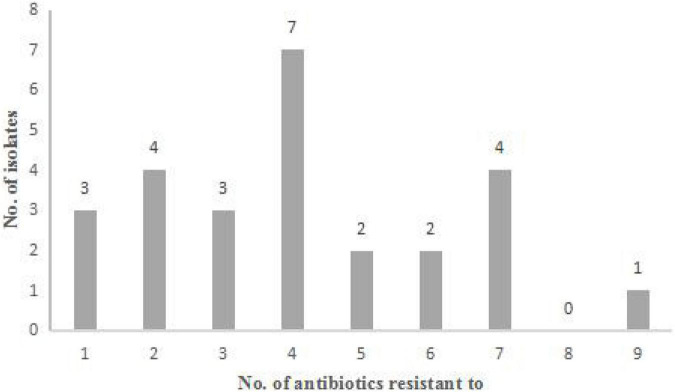
Antibiotic resistance characteristics of 26 *Staphylococcus aureus* strains. Distribution of the number of antibiotics that the strains exhibit resistance.

A total of 125 raw milk samples were collected from 125 dairy farms located in China, including 25 goat milk samples from Shaanxi province, 25 goat milk samples from Shandong province, 25 camel milk samples from Xinjiang Uygur Autonomous Region, 25 buffalo milk samples from Guangxi Zhuang Autonomous Region, and 25 yak milk samples from Sichuan province, from April to June 2016 (Figure 1). All samples were collected from healthy animals by mechanical milking. The milking transferred into sterile bottles and transported immediately to laboratory at 4°C, for bacteriological analysis.

### Isolation and Identification of *Staphylococcus aureus*

To isolate and detect the *S. aureus*, each sample was diluted 10-fold in sterile peptone water and homogenized. Aliquots (1 mL) were placed onto Baird-Parker agar supplemented with 5% egg yolk and tellurite (Beijing Land Bridge Technology Ltd., Beijing, China). The plates were incubated for 24–48 h at 37°C. Colonies with typical black appearance and surrounded by a clear zone were enumerated as *S. aureus*.

Presumptive colonies were confirmed by polymerase chain reaction (PCR) (Bio-Rad S1000, United States) detection of the thermonuclease gene (*nuc*, *S. aureus* specific) (Supplementary Table 1). DNA of the strains was extracted using the InstaGene Matrix DNA extraction kit (Bio-Rad Laboratories, Hercules, California, United States) following the manufacturer’s instructions. The amplification conditions and reagents for the PCR assays were those described by Liu et al. (2017). Negative control (without DNA template) and positive control (*S. aureus* ATCC 6538) templates were included in all PCR assays. After identification, one to three colonies per sample were randomly selected for subsequent analysis. All strains were stored with sterile magnetic beads at −80°C until further analysis.

### Antimicrobial Resistance Screening

Antimicrobial resistance of *S. aureus* were determined by the agar disc diffusion method according to Clinical and Laboratory Standards Institute [CLSI] (2018a,b). According to the frequency of antibiotical therapy for dairy mastitis in China, the antimicrobials used for susceptibility testing included 13 antimicrobial agents: Penicillin G (10U), amoxicillin-clavulanic acid (20/10 μg), tetracycline (30 μg), gentamicin (10 μg), oxacillin (1 μg), streptomycin (10 μg), cefoxitin (30 μg), erythromycin (15 μg), ampicillin (10 μg), chloramphenicol (30 μg), kanamycin (30 μg), clindamycin (2μg), and trimethoprim-sulfamethoxazole (1.25/23.75 μg) were used as antimicrobial agents (Oxoid, Basingstoke, United Kingdom). *Staphylococcus aureus* ATCC 6538 and *E. coli* ATCC 25922 were used as quality controls. The antimicrobial resistance experiment of *S. aureus* was repeated twice.

### Antimicrobial Resistance Genes

All resistant strains were screened for antimicrobial resistance genes by PCR amplification. The information of all primers is shown in Supplementary Table 1. The primers were synthesized by Sangon Biotech Co., Ltd. (Shanghai, China). The genes encoding penicillin (*bla*Z and *mec*A), cefoxitin (*cfx*A and *mec*A), aminoglycoside [*aac6*′-*aph2″*, *ant(6)-*Ia, *ant(4′)-Ia*], chloramphenicol (*fex*A and *cat*A), tetracycline [*tet*(K), *tet*(L), *tet*(M) and *tet*(O)], erythromycin [*erm*(A), *erm*(B), *erm*(C), *msr*(A) and *msr*(B)], streptomycin [*ant(6)-*Ia], and oxacillin (*mec*A) resistance genes were detected by PCR. PCR products were electrophoresed in a 1.5% (m/v) agarose gels (Hydragene) with SYBR Safe DNA Stain (Invitrogen).

### Detection of Virulence-Associated Genes

The detection of genes encoding staphylococcal enterotoxins (*sea*, *seb*, *sec*, *sed*, *see*, *seg*, *seh*, *sei*, *sek*, *sel*, *ser*, *ses sej*, and *set*), enterotoxin-like (*selm*, *selo*, *selp*, and *selu*), toxic-shock syndrome toxin (*tst*-1), exfoliative toxin genes (*eta* and *etb*), and Panton-Valentine leukocidin (*pvl*) was performed by PCR according to Wang et al. (2016). The target genes, primer sequences, and target fragment of PCR products are given in Supplementary Table 1.

### Statistical Analysis

The data of prevalence and gene distribution between different categories of raw milks were analyzed using χ^2^-test and Fisher’s exact test with *P* < 0.05 considered as statistically significant in SPSS software (ver. 19.0, IBM Corp., Armonk, NY, United States).

## Results

### Prevalence of *Staphylococcus aureus*

Out of 125 samples, 36 strains of *S. aureus* were isolated, including 10 strains (40%) of 25 goat milk samples from Shaanxi province, 6 strains (24%) of 25 goat milk from Shandong province, 9 strains (36%) of 25 buffalo milk samples from Guangxi Zhuang Autonomous Region, and 6 strains (24%) of 25 camel milk samples from Xinjiang Uygur Autonomous Region and 5 strains (20%) of 25 yak milk samples from Sichuan province.

### Antimicrobial Susceptibility Testing

The 36 strains of *S. aureus* were tested for susceptibility against 13 antimicrobial agents by the disc diffusion method. A total of 26 strains (26/36, 72.2%) showed antibiotics resistance. 19 strains (19/36, 52.8%) showed resistance to at least three antimicrobial classes. Moreover, 4 strains (4/36, 11.1%) were resistant to 7 antibiotics tested, and 1 strain (1/36, 2.8%) was resistant to 9 antibiotics tested (Figure 1). Antimicrobial resistance was most frequently observed to penicillin G (18/36, 50%), followed by to tetracycline (15/36, 41.7%), gentamicin (13/36, 36.1%), ampicillin (12/36, 33.3%), erythromycin (9/36, 25%), cefoxitin (8/36, 22.2%), streptomycin (8/36, 22.2%), kanamycin (7/36, 19.4%), chloramphenicol (6/36, 16.7%), oxacillin (6/36, 16.7%), sulfamethoxazole-trimethoprim (3/36, 8.3%), clindamycin (1/36, 2.8%), and amoxicillin-clavulanic acid (0/36, 0) (Table 1). Among the 36 *S. aureus*, the antimicrobial resistance was frequently observed in *S. aureus* from goat milks (13/36, 36.1%), followed by buffalo milks (6/36, 16.7%), camel milks (4/36, 11.1%), and yak milks (3/36, 8.3%).

**TABLE 1:** Antibiotic resistance of *Staphylococcus aureus* strains isolated from different raw milks.

Antibiotic class	Antibiotic	Number of positive strains for animal species (%)				Total number of positive strains (%)	χ ^2^	*P*
		Goat	Buffalo	Camel	Yak			
β-lactams	Penicillin G	10/16(62.5)	3/9(33.3)	3/6(50)	2/5(40)	18/36(50)	2.281	0.541
	Ampicillin	7/16(43.8)	2/9(22.2)	2/6(33.3)	1/5(20)	12/36(33.3)	1.615	0.708
	Cefoxitin	4/16(25)	2/9(22.2)	1/6(16.7)	1/5(20)	8/36(22.2)	0.433	1.000
	Oxacillin	6/16(37.5)	0/9(0)	0/6(0)	0/5(0)	6/36(16.7)	6.609	0.0418*
Macrolides	Erythromycin	6/16(37.5)	2/9(22.2)	1/6(16.7)	0/5(0)	9/36(25)	2.715	0.466
Lincomycin	Clindamycin	1/16(6.3)	0/9(0)	0/6(0)	0/5(0)	1/36(2.8)	2.156	1.000
Sulfonamides	Sulfamethoxazole-trimethoprim	1/16(6.3)	1/9(11.1)	1/6(16.7)	0/5(0)	3/36(8.3)	1.638	0.849
Tetracyclines	Tetracycline	10/16(62.5)	1/9(11.1)	2/6(33.3)	2/5(40)	15/36(41.7)	6.431	0.087
Aminoglycosides	Kanamycin	4/16(25)	2/9(22.2)	1/6(16.7)	0/5(0)	7/36(19.4)	1.372	0.869
	Gentamicin	7/16(43.8)	3/9(33.3)	2/6(33.3)	1/5(20%)	13/36(36.1)	1.034	0.856
	Streptomycin	5/16(31.3)	2/9(22.2)	1/6(16.7)	0/5(0)	8/36(22.2)	1.901	0.654
Chloramphenicol	Chloramphenicol	5/16(31.3)	1/9(11.1)	0/6(0)	0/5(0)	6/36(16.7)	3.427	0.247

“*” indicates that the difference is statistically significant (P < 0.05).

Moreover, six strains isolated from goats were identified as methicillin-resistant *S. aureus* (MRSA). Only one *S. aureus*, which was isolated from goat milk, showed resistance to clindamycin. There was no sulfamethoxazole-trimethoprim-resistant, kanamycin-resistant, and streptomycin-resistant strains isolated from yak milk samples, and there was no chloramphenicol-resistant strains isolated from yak and camel milk samples.

### Screening of Antimicrobial Resistance Genes

**FIGURE 2:**
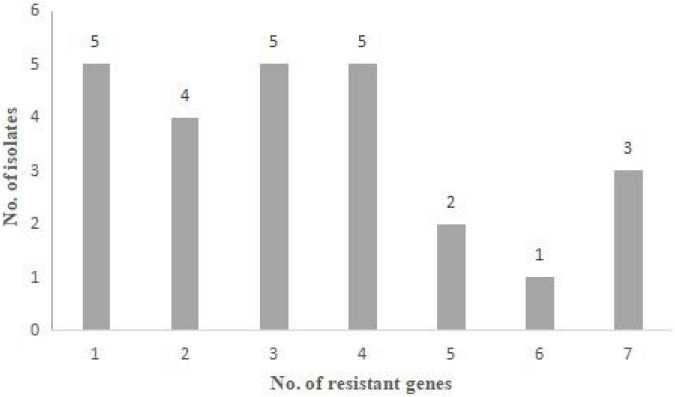
Antibiotic resistance genes of 26 *Staphylococcus aureus* strains. Distribution of the number of antibiotic resistance genes that the strains harbored.

The results of antimicrobial resistance genes detection are presented in Figure 2 and Tables 2, 3. Among the 26 resistant strains, 96.2% of strains (25/26) harbored at least one resistant gene (Figure 2).

**TABLE 2:** Antimicrobial resistance genes identified in *S. aureus* from different raw milks.

Resistance gene	Number of positive strains for animal species (%)				Total number of positive strains (%)	χ ^2^	*P*
	Goat	Buffalo	Camel	Yak			
*bla*Z	10/16(62.5%)	3/9(33.3%)	3/6(50%)	2/5(40%)	18/26(69.2%)	1.792	0.710
*mec*A	6/16(37.5%)	0/9(0)	0/6(0)	0/5(0)	6/26(23.1%)	5.887	0.074
*cfx*A	3/16(18.8%)	2/9(22.2%)	1/6(16.7%)	0/5(0)	6/26(23.1%)	5.887	0.074
*ant*(4′)-*Ia*	3/16(18.8%)	2/9(22.2%)	1/6(16.7%)	0/5(0)	6/26(23.1%)	1.010	1.000
*aac*6′-*aph*2″	7/16(43.8%)	3/9(33.3%)	2/6(33.3%)	1/5(20%)	13/26(50.0%)	0.709	1.000
*ant(6)-*Ia	1/16(6.3%)	0/9(0)	0/6(0)	0/5(0)	1/26(3.9%)	–	–
*fex*A	2/16(12.5%)	0/9(0)	0/6(0)	0/5(0)	2/26(7.7%)	1.692	1.000
*cat*A	0/16(0)	0/9(0)	0/6(0)	0/5(0)	0/26(0)	–	–
*tet*(M)	9/16(56.3%)	0/9(0)	0/6(0)	1/5(20%)	10/26(38.5%)	10.855	0.004*
*tet*(L)	6/16(37.5%)	1/9(11.1%)	0/6(0)	0/5(0)	7/26(26.9%)	4.095	0.215
*tet*(K)	0/16(0)	0/9(0)	2/6(33.3%)	0/5(0)	2/26(7.7%)	6.822	0.028*
*tet*(O)	0/16(0)	0/9(0)	0/6(0)	1/5(20%)	1/26(3.9%)	5.114	0.115
*erm*(A)	0/16(0)	0/9(0)	0/6(0)	0/5(0)	0/26(0)	–	–
*erm*(B)	2/16(12.5%)	0/9(0)	1/6(16.7%)	0/5(0)	3/26(11.5%)	1.990	0.820
*erm*(C)	4/16(25%)	2/9(22.2%)	0/6(0)	0/5(0)	6/26(23.1%)	2.222	0.589
*msr*(A)	0/16(0)	0/9(0)	0/6(0)	0/5(0)	0/26(0)	–	–
*msr*(B)	1/16(6.3%)	2/9(22.2%)	1/6(16.7%)	0/5(0)	4/26(15.4%)	2.926	0.329

“–” indicates no such value; “*” indicates that the difference is statistically significant (P < 0.05).

**TABLE 3:** Distribution of antibiotic-resistance genes among resistant *Staphylococcus aureus.*

Antibiotics	Resistance genes	No. (%) of positive strains
Penicillin G	*blac*Z	18/18(100%)
Cefoxitin	*cfx*A	6/8(75%)
Aminoglycosides	*aac*6′-*aph*2″	10/17(58.8%)
	*ant(6)-*Ia	0/17(0%)
	*ant(4′)-Ia*	2/17(11.8%)
	*aac*6′-*aph*2″ + *ant(6)-*Ia	1/17(5.9%)
	*aac*6′-*aph*2″ + ant(4′)-Ia	4/17(23.5%)
Chloramphenicol	*fex*A	2/6(33.3%)
	*cat*A	0/6(0)
Tetracycline	*tet(*K)	2/15(13.3%)
	*tet*(L)	2/15(13.3%)
	*tet*(M)	5/15(33.3%)
	*tet*(O)	1/15(6.7%)
	*tet*(M) + *tet*(L)	5/15(33.3%)
Erythromycin	*erm*(A)	0/9(0)
	*erm*(B)	1/9(11.1%)
	*erm*(C)	2/9(22.2%)
	*msr*(A)	1/9(11.1%)
	*msr*(B)	0/9(0)
	*erm*(B) + *erm*(C)	1/9(11.1%)
	*erm*(C) + *msr*(B)	3/9(33.3%)
	*erm*(B) + *msr*(B)	1/9(11.1%)
Oxacillin	*mec*A	6/6(100%)

As shown in Supplementary Table 2, the antimicrobial resistance genes were frequently detected in *S. aureus* from goat milks (13/13, 100%), camel milks (4/4, 100%) and yak milks (3/3, 100%). The most predominant resistance genes were *bla*Z (18/26, 69.2%), *aac6*′-*aph2″*(13/26, 50.0%), *tet*(M) (10/26, 38.5%), *tet*(L) (7/26, 26.9%), *mec*A (6/26, 23.1%), *erm(*C) (6/26, 23.1%), *ant(4′)-Ia* (6/26, 23.1%), *cfx*A (6/26, 23.1%), *msr*(B) (4/26, 15.4%), *erm*(B) (3/26, 11.5%), *tet*(K) (2/26, 7.7%), *fex*A (2/26, 7.7%), *tet*(O) (1/26, 3.9%), and *ant(6)-*Ia (1/26, 3.9%). The *cat*A, *erm*(A) and *msr*(A) genes were not detected in *S. aureus* (Table 2). Moreover, the *mec*A, *ant(6)-Ia* and *fex*A gene were only detected in the *S. aureus* from goat milk. The *tet*(K) and *tet*(O) genes were only detected in camel and yak milks, respectively.

Moreover, all the penicillin G-resistant *S. aureus* carried *bla*Z gene, and all the oxacillin-resistant *S. aureus* carried *mec*A gene. The aminoglycosides-resistant strains (*n* = 17) harbored one or more resistant genes, including *aac*6′-*aph*2″ (10/17, 58.8%), *ant(4′)-Ia* (2/17, 11.8%), *aac*6′-*aph*2″ + *ant*(4′)-*Ia* (4/17, 23.5%), *aac*6′-*aph*2″ and *ant(6)-Ia* (1/17, 5.9%). The tetracycline-resistant strains (*n* = 15) harbored one or more resistant genes, including *tet*(M) + *tet*(L) (5/15, 33.3%), *tet*(M) (5/15, 33.3%), *tet*(K) (2/15, 13.3%), *tet*(L) (2/15, 13.3%), and *tet*(O) (1/15, 6.7%). The erythromycin-resistant strains (*n* = 9) harbored one or more genes, including *erm*(B), *erm(*C), *msr*(A), and *msr*(B). The *erm*(A) genes were not detected in erythromycin-resistant strains. The *msr*(B) gene was detected together with *erm*(B) and *erm*(C) in the same strains.

### Distribution of Virulence Genes

As shown in Table 4, the predominant toxin genes were *sec* (8/26, 30.8%), followed by *sea* (3/26, 11.5%), *seg* (3/26, 11.5%), *tst* (3/26, 11.5%), *sel* (2/26, 7.7%), *sed* (1/26, 3.9%), *sei* (1/26, 3.9%), *sej* (1/26, 3.9%), and *pvl* (1/26, 3.9%). Thirteen virulence genes, including *seb*, *see*, *seh*, *sek*, *ser*, *ses, set, selm*, *selo*, *selp*, *selu, eta*, and *etb*, were not detected in *S. aureus*. Moreover, 42.3% of the strains (11/26) harbored at least one virulence gene. Among the 26 of *S. aureus*, 6 strains from goat milk samples harbored 17 virulence genes, 1 strain from buffalo milk samples harbored 1 virulence gene, 2 strains from camel milk samples harbored 3 virulence genes, and 2 strains from yak milk samples harbored 2 virulence genes.

**TABLE 4:** Virulence genes identified in *Staphylococcus aureus* strains isolated from different raw milk samples (*n* = 26).

Class of toxin	Tpye of toxin	Gene	Number of positive strains for animal species (%)				Total number of positive strains (%)	χ ^2^	*P*
			Goat	Buffalo	Camel	Yak			
Enterotoxins	Enterotoxin A	*sea*	2/13(15.4%)	0/6(0%)	0/4(0%)	1/3(33.3%)	3(11.5%)	2.565	0.470
	Enterotoxin C	*sec*	5/13(38.5%)	1/6(16.7%)	2/4(50%)	0/3(0%)	8(30.8%)	2.578	0.485
	Enterotoxin D	*sed*	1/13(7.7%)	0/6(0%)	0/4(0%)	0/3(0%)	1(3.9%)	2.181	1.000
	Enterotoxin G	*seg*	2/13(15.4%)	0/6(0%)	0/4(0%)	1/3(33.3%)	3(11.5%)	2.565	0.470
	Enterotoxin I	*sei*	1/13(7.7%)	0/6(0%)	0/4(0%)	1/5(20%)	1(3.9%)	2.181	1.000
	Enterotoxin J	*sej*	1/13(7.7%)	0/6(0%)	0/4(0%)	0/3(0%)	1(3.9%)	2.181	1.000
	Enterotoxin L	*sel*	2/13(15.4%)	0/6(0%)	0/4(0%)	0/3(0%)	2(7.7%)	1.692	1.000
Toxic-shock syndrome toxin	Toxic-shock syndrome toxin	*tst-1*	3/13(23.1%)	0/6(0%)	0/4(0%)	0/3(0%)	3(11.5%)	4.551	0.265
Paton-valentine leukocidin	Paton-valentine leukocidin	*pvl*	0/13(0%)	0/6(0%)	1/4(25%)	0/3(0%)	1(3.9%)	4.538	0.269

## Discussion

*Staphylococcus aureus* is one of the main pathogens in raw milk and dairy products, which may lead to economic losses (Aires-de-Sousa et al., 2007; Stapels et al., 2014). *Staphylococcus aureus* has a strong capability to produce a wide variety of enterotoxins (Liu et al., 2017). The enterotoxins produced by *S. aureus* are common causes of bovine mastitis pathogenesis and food-borne illnesses (Wang et al., 2016). However, little is known about the presence of *S. aureus* in goat, buffalo, camel, and yak milk in China. In the present study, the prevalence, antimicrobial susceptibility, and key virulence genes of *S. aureus* isolated from goat, buffalo, camel, and yak milk samples in China has been assessed.

The prevalence of *S. aureus* in different raw milk samples varies depending on the country. In our study, raw buffalo milk samples showed the highest prevalence of *S. aureus* (9/25, 36%), followed by goat milk (16/50, 32%), camel milk (6/25, 24%), and yak milk (5/25, 20%). These results are significantly higher than that in previous reports which have shown that the occurrence rate of *S. aureus* in buffalo milk was 17.5%, followed by goat milk (7.5%) and camel milk (3.4%) in Iran (Ebrahim and Forough, 2013), 31.43% in goat milk in Egypt (Elbagory, 2017), and 15% in camel milk in Sudan (Shuiep et al., 2009). In contrast, much higher occurrence rate of *S. aureus* was 43.1% in goat milk in Northern Italy (Cortimiglia et al., 2015), 54.02% in camel milk in Pakistan (Aqib et al., 2017), 47.36% in yak milk in India (Biswas et al., 2014), and 32% in camel milk in Kenya (Njage et al., 2013). Overall, the results indicate that *S. aureus* is common in different raw milks, including goat, buffalo, camel and yak milk in China. This suggests that more attention should be paid to explore the method of controlling *S. aureus* occurrence in goat, buffalo, camel, and yak milk.

The antibiotics resistance has correlation to many factors, including excessive use of antibiotics, spread of resistance, and the background antibiotic resistance levels (Hu et al., 2014; Low et al., 2016; Crofts et al., 2017). The poorly regulated use of antibiotics on dairy animals in the different raw milk production systems examined may be an important reason for the high antimicrobial resistance. In this study, the antimicrobial resistance was the frequently observed in *S. aureus* from goat milk (36.1%) and buffalo milks (16.7%). The results indicated that the antibiotics resistance of *S. aureus* isolated from goat milk in Shandong and Shaanxi province, buffalo milk in Guangxi Zhuang Autonomous Region were very serious in China, although less than raw cow milk (Liu et al., 2017). A high emission densities of the antibiotics in China were reported in Shandong province, Shaanxi province, and Guangxi Zhuang Autonomous Region (Zhang et al., 2015). It implied that the high antimicrobial resistance of *S. aureus* from goat and buffalo milk may be related to it.

In the current study, *S. aureus* showed a high percentage of antimicrobial resistance to penicillin G, tetracycline, gentamicin, and ampicillin. Similar findings were reported by Oliveira et al. (2011), who found that 100% of *S. aureus* from buffalo milks were resistant to penicillin G and ampicillin, and by Rola et al. (2015), who found that coagulase-positive staphylococcus isolated from goat milk were resistant to penicillin G and tetracycline, and by Njage et al. (2013), who found that *S. aureus* isolated from camel milk from Kenya showed a high percentage of antimicrobial resistance to ampicillin, gentamicin, and tetracycline. Moreover, a total of 26 strains (26/36, 72.2%) showed antibiotics resistance, and 19 strains (19/26, 52.8%) showed resistance to at least three antimicrobial classes in our study. Based on the high percentage of resistance, these antibiotics should be used with caution for mastitis caused by *S. aureus* in goat, buffalo, and camel milk in China.

MRSA has been identified as an emerging pathogen in livestock animals that is readily transferable to humans in contact with livestock (Liu et al., 2017). Numerous reports have described the prevalence of MRSA in bovine milk (Caruso et al., 2016; Basanisi et al., 2017), and the transmission of MRSA between people working on farms and dairy cattle (Antoci et al., 2013; Mole, 2013). On the contrary, little is known about the prevalence of MRSA in other ruminants (e.g., goats, camels, buffalo, yaks), although the consumption of milk from different animals species is more and more common. In the present study, 16.7% of *S. aureus* (6/36) isolated from goat milk were identified as methicillin-resistant strains (MRSA). The results showed that there is a high prevalence in goat milks. The prevalence of MRSA in Southern Italy was 1.23% in goat milk and sheep bulk tank milk (Caruso et al., 2016), 8.3% in cow milk samples and dairy products (Basanisi et al., 2017). Moreover, there was no MRSA strains detected in buffalo, yak, and camel milk samples in our study. There was difference in data regarding the prevalence of MRSA strains in buffalo milk samples. Pamuk et al. (2012) reported that 2.5% of the buffalo samples were contaminated with MRSA in Turkey. El-Ashker et al. (2015) found that 24.5% of *S. aureus* in buffalo milk were identified as MRSA in Dakahlia Governorate, Egypt.

In the present study, 42.3% of the strains (11/26) harbored at least one virulence genes, which was much lower than reported in other studies. Liu et al. (2017) found that 60.3% of *S. aureus* from cow milk harbored one or more virulence genes. Martins et al. (2017) found that 62.5% of *S. aureus* from sheep milk samples showed some toxin-encoding genes. There are also reports showing that 67% of the detected *S. aureus* exhibited at least one enterotoxin gene, many caprine, or ovine raw milk products may be contaminated with low levels of enterotoxigenic *S. aureus* (Merz et al., 2016). Liu et al. (2019) reported that 62 *S. aureus* (72.5%) carried virulence genes, including *set, hlb, hld, lukED, ebp, clfA, and clfB.* Moreover, the largest percentage of *sec* (31.8%) genes was found in our study. Several authors have also reported that enterotoxin genes *sea* and *sec* are the most common in *S. aureus* isolated from raw milk (Carfora et al., 2015; Liu et al., 2017). *Staphylococcus aureus* from different kinds of milk samples exhibited different virulence gene patterns. For goat milk, *sec* gene was the most frequent in the study, and also reported as the most common virulence genes in Brazil (Lyra et al., 2013; Cavicchioli et al., 2015; Salaberry et al., 2015). Other researchers have reported that the most prevalent enterotoxin genes were *sec* and *sel* from goat milk samples in Switzerland (Merz et al., 2016). For camel milk, the *sec* and *pvl* genes have been detected in the study. Njage et al. (2013) found that *pvl* gene was detected in *S. aureus* from raw and fermented camel milk from Kenya and Somali. However, three *S. aureus* from camel milk were positive for *sec*, *seg*, *sei*, *sem*, *sen*, and *seo*, respectively (Shuiep et al., 2009). Majority of *S. aureus* were co-carried many virulence genes in goat, yak, camle, and bufflo milk, and it may pose great potential risk to public health. In order to estimate the actual impact on public health, it was necessary to the detection of virulence genes in goat, buffalo, and camel milk samples.

## Conclusion

In conclusion, the antimicrobial resistance and virulence genes of *S. aureus* in goat, buffalo, camel, and yak milk in China were assessed. Our data indicated that *S. aureus*, which exhibit a high percentage of antimicrobial resistant and carried multiple virulence genes, were commonly found in different raw milks, especially goat milk. Although the prevalence of MRSA in raw milk from china was low, MRSA may pose a potential threat for consumers of raw milk or dairy products made with raw milk and for people working or living in close contact with animals. Therefore, it was very important to monitor the usage of antimicrobial agents and the potential transfer of antimicrobial resistance genes in goat milk.

## Data Availability Statement

The original contributions presented in the study are included in the article/Supplementary Material, further inquiries can be directed to the corresponding author/s.

## Author Contributions

HL, LD, and YZ designed and performed the researches. LM helped with the data analysis. CW and JW gave advices to the researchers. NZ gave the opinions on the researches design. All authors contributed to the article and approved the submitted version.

## Conflict of Interest

The authors declare that the research was conducted in the absence of any commercial or financial relationships that could be construed as a potential conflict of interest.

## Publisher’s Note

All claims expressed in this article are solely those of the authors and do not necessarily represent those of their affiliated organizations, or those of the publisher, the editors and the reviewers. Any product that may be evaluated in this article, or claim that may be made by its manufacturer, is not guaranteed or endorsed by the publisher.
